# Fast determination of the optimal rotational matrix for macromolecular superpositions

**DOI:** 10.1002/jcc.21439

**Published:** 2009-12-16

**Authors:** Pu Liu, Dimitris K Agrafiotis, Douglas L Theobald

**Affiliations:** 1 Johnson & Johnson Pharmaceutical Research and Development, L.L.C.665 Stockton Drive, Exton, Pennsylvania 19341; 2 Department of Biochemistry, Brandeis University415 South Street, Waltham, Massachusetts 02454-9110

**Keywords:** rotational matrix, superposition, RMSD, quaternion, adjoint matrix, root mean squared deviation, protein structure alignment, fragment-assembly, conformational sampling

## Abstract

Finding the rotational matrix that minimizes the sum of squared deviations between two vectors is an important problem in bioinformatics and crystallography. Traditional algorithms involve the inversion or decomposition of a 3 × 3 or 4 × 4 matrix, which can be computationally expensive and numerically unstable in certain cases. Here, we present a simple and robust algorithm to rapidly determine the optimal rotation using a Newton-Raphson quaternion-based method and an adjoint matrix. Our method is at least an order of magnitude more efficient than conventional inversion/decomposition methods, and it should be particularly useful for high-throughput analyses of molecular conformations. © 2009 Wiley Periodicals, Inc. J Comput Chem, 2010

## Introduction

The root-mean-square distance (RMSD) is a common metric used to characterize the similarity between two vector sets (e.g., protein structures).^1^ The minimum RMSD is conventionally determined using the method of least squares in which an optimal translation vector and rotation matrix are found that minimize the sum of the squared distances between corresponding atoms in two coordinate sets. Determining the optimal rotation matrix can be a rate-limiting step in several computationally intensive structural bioinformatics algorithms where large numbers of structures must be compared, such as in aligned-fragment-pair multiple protein structure alignment,^2^–^4^ fragment-assembly protein structure predictions,^5^ conformation sampling for structure-based drug design,^6^ and high-throughput superpositioning of analogous and homologous protein domains in the entire PDB database.^7^ Hence, more efficient superposition algorithms are desirable.

Considerable effort has been directed toward developing fast and robust algorithms for determining the RMSD and the corresponding optimal rotation.^8^–^15^ For example, Kabsch calculates the optimal rotation by solving a least-squares problem with orthogonality constraints ensured by a Lagrange multiplier. This method requires the calculation of the eigenvalues and eigenvectors of a 3 × 3 matrix. In addition, improper rotation matrices may arise when the determinant of a key matrix is negative,^11^ which requires special handling.^16^–^18^ Ferro and Hermans (1977) approximate the rotational matrix by applying the best rotation about each Cartesian axis iteratively, which requires expensive square root operations and matrix multiplications.^9^ McLachlan describes a method to calculate the rotational matrix using conjugate gradient minimization and a succession of finite rotations about the conjugate axes.^13^ The coordinate sets must be updated in every iteration making this method computationally expensive for large systems. Lesk reduces the superposition problem to an unconstrained maximization of a function of a single variable. However, the evaluation of this function requires dynamically updating the coefficients of a quartic polynomial and locating its real roots.^12^

Horn,^10^ Diamond,^8^ Kearsley,^15^ and Theobald^14^ represent the rotations as quaternions and cast the original problem as an eigenvalue/eigenvector problem for a 4 × 4 matrix. In particular, Diamond developed a fast iterative method to calculate the minimum RMSD. However, his method is unstable when the required rotation is close to 180^o^ because the matrix to be inverted becomes singular.^8^,^14^ Theobald circumvents the decomposition and inversion problem by using a Newton-Raphson (NR) algorithm that solves the characteristic polynomial for the minimum RMSD. While Theobald's method does not provide the optimal rotation matrix, the approach is over an order of magnitude more efficient when only the RMSD is of interest.^14^

**Table 1:** Comparison of the Average Computational Time Required to Determine One Optimal Rotational Matrix for the Current Method (QCP) and the Traditional Household Reduction and QL Decomposition Approach (H-QL)

Protein	PDB Id	Number of residues	Number of structures	Time (μs) QCP	Time (μs) H-QL
d-Galactose/Glucose binding protein	2GBP	309	297	0.185	3.57
Human CDC25B catalytic domain	1QB0	177	400	0.200	3.54
Barstar	1A19	89	191	0.201	4.11
Alpha-Amylase inhibitor	1HOE	74	129	0.200	4.37
Calmodulin	1CFD	72	196	0.195	3.96
Ferredoxin II	1FXD	58	141	0.196	3.92

Based on Horn's quaternion approach and Theobald's NR quaternion-based characteristic polynomial (NR-QCP) method, we present an extremely efficient algorithm to determine the optimal rotational matrix in the superposition problem. As in the previous article,^14^ the RMSD is first evaluated by solving for the most positive eigenvalue of the key matrix using the NR-QCP algorithm. Here, we show how to use this eigenvalue to rapidly determine the optimal rotation matrix. The best rotation is given by the corresponding eigenvector, which is calculated via the adjoint matrix. The present method has several advantages: (i) the time required to calculate the rotation matrix is independent of the system size after a special 3 × 3 matrix is constructed from the coordinates, (ii) no special cases need to be handled separately, and (iii) the approach is extremely fast, straightforward, and robust, as there is no expensive matrix inversion or decomposition. To our knowledge, the algorithm presented here is by far the fastest method currently available for superpositioning macromolecules.

## The Weighted Least-Squares Superposition Problem

The structure of a molecule with *N* atoms can be conveniently represented as a *N* × 3 matrix in which the *i*-th row corresponds to the *x,y,z* coordinates of the *i*-th atom. Let ***A*** and ***B*** be two structures under consideration, and ***W*** be a diagonal weighting matrix with the *i*-th diagonal element representing the weight for the *i*-th atom. If each structure is translated so that its centroid is at the origin, the superposition problem is to find an optimal rotation ***R*** that minimizes the following function^11^,^19^:

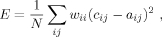
(1)
where ***C** = **BR***; *c_ij_* and *a_ij_* are the elements of the matrices ***C*** and ***A***, respectively, and *w_ii_* is the *i*-th diagonal element of the matrix ***W***.

If eq. (1) is expressed in matrix format and expanded, it can be seen that:

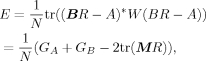
(2)
where tr(***X***) is the trace of the matrix ***X*,** X* represents the transpose of **X**, *G*_*A*_ is the weighted inner product of structure **A**,

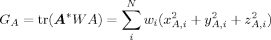
(3)
and the matrix ***M*** is the inner product of two structures **A** and **B**,

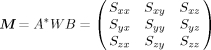
(4)
and 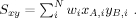


## Determination of the Optimal Rotation Matrix

Horn has shown that the optimal rotational matrix in the unit quaternion representation is the eigenvector associated with the most positive eigenvalue of the following symmetric 4 × 4 matrix ***K***^10^:

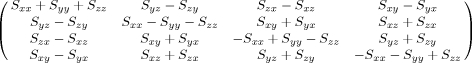


The eigenvalues can be determined by locating the roots of the characteristic polynomial det(***K** − λ**I***), where ***I*** is the identity matrix, λ is one of the eigenvalues, and det(***X***) represents the determinant of the matrix ***X***. As shown by Theobald,^14^ the coefficients of the quartic polynomial for the key matrix ***K*** can be determined with at most 66 floating point operations (FLOPs). For this 4 × 4 matrix, the most positive root is bounded from above by the average of two self inner products, (*G*_*A*_ + *G*_*B*_)/2. The use of this upper bound as the initial guess leads to quick and stable location of the most positive root with the NR method.^14^ This method only takes about five iterations for convergence to a relative precision of 10^−6^.^14^ Because there are only 11 FLOPs involved in every iteration,^14^ this method is extremely efficient in calculating the most positive root from which the RMSD is given by 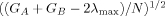.

The optimal rotation matrix corresponds to the eigenvector associated with the largest eigenvalue of the key matrix ***K.*** As the eigenvalue has been determined as stated earlier, one may solve for the eigenvector using standard iterative eigen-decomposition methods to solve the homogeneous equation (***K*** − λ***I***)**e** = 0. However, because ***K*** is a small 4 × 4 matrix, one may efficiently determine the eigenvector analytically from the adjoint matrix. From basic linear algebra, it can be shown that ***X*** adj(***X***) = det(***X***)***I***, where adj(***X***) is the adjoint matrix for any matrix ***X***.^20^ If ***X*** = ***K*** − λ***I*** and λ is an eigenvalue (i.e., det(***K*** − λ***I***) = 0), then any nonzero column of the adjoint of the matrix (***K*** − λ***I***) is an eigenvector associated with the eigenvalue λ.^20^ Calculating the first column of the adjoint matrix requires only 28 multiplications and 26 subtractions/additions. If the first column of the adjoint matrix is zero or very small, then calculation of the eigenvector may suffer from floating point error, and the calculation of one or more columns is necessary. However, for all the >10^9^ superposition operations we performed, we have found that the first column is sufficient. Even in the worst case, where the entire adjoint matrix needs to be constructed, only an additional 60 multiplications and 39 subtractions/additions are required. The optimal rotational matrix is then uniquely determined by the resulting unit quaternion.

To explore the robustness and efficiency of this method, we performed >10^9^ superpositions for short protein fragments. Pairwise RMSDs were also calculated for protein conformations from the publicly accessible “ensemble protein database.”^21^ Table 1 compares the times for determining the optimal rotation determination using our approach QCP versus the traditional Householder reduction method followed by QL decomposition with implicit shift (H-QL).^22^,^23^ The time spent for the construction of the matrix ***M*** is not included in timing because it is a prerequisite for all the methods. For accurate timing, the rotational matrix was calculated repeatedly 500,000 and 50,000 times for the QCP and H-QL approaches, respectively. All calculations were performed on an IBM Thinkpad T61 laptop computer equipped with a single dual-core 2GHz mobile Intel processor and 1.96 GB 667MHz DRAM. Our QCP method is about 20 times faster than the H-QL method, while giving identical rotational matrices within floating point error. Many widely used programs rely on extensive superpositioning. For example, FATCAT^4^ and Matt^2^ were proven to be able to align multiple protein structures and identify homologous residues efficiently. Rosetta^5^ has widely used in *ab initio* protein prediction and protein design.^24^,^25^ These programs could all potentially benefit from the algorithm presented herein. For the convenience of the audience, ANSI C source code of the present algorithm is organized to be integrated into existing packages straightforwardly with minimal effort. The code and the instruction are publicly available without charge under the BSD license from http://theobald.brandeis.edu/QCP/. For questions regarding to the code, please contact pliu24@its.jnj.com or dtheobald@brandeis.edu.
